# Accelerated mafic weathering in Southeast Asia linked to late Neogene cooling

**DOI:** 10.1126/sciadv.adf3141

**Published:** 2023-03-29

**Authors:** Germain Bayon, Martin Patriat, Yves Godderis, Anne Trinquier, Patrick De Deckker, Denise K. Kulhanek, Ann Holbourn, Yair Rosenthal

**Affiliations:** ^1^ Univ Brest, CNRS, Ifremer, Geo-Ocean, F-29280 Plouzané, France.; ^2^ Géosciences-Environnement Toulouse, CNRS-Université Paul Sabatier, F-31400 Toulouse, France.; ^3^ The Australian National University, Research School of Earth Sciences, Canberra, ACT 2601, Australia.; ^4^ Institute of Geosciences, Christian-Albrechts-University of Kiel, 24118 Kiel, Germany.; ^5^ Department of Marine and Coastal Sciences and Department of Earth and Planetary Sciences, Rutgers, State University of New Jersey, New Brunswick, NJ 08901, USA.

## Abstract

Arc-continent collision in Southeast Asia during the Neogene may have driven global cooling through chemical weathering of freshly exposed ophiolites resulting in atmospheric CO_2_ removal. Yet, little is known about the cause-and-effect relationships between erosion and the long-term evolution of tectonics and climate in this region. Here, we present an 8-million-year record of seawater chemistry and sediment provenance from the eastern Indian Ocean, near the outflow of Indonesian Throughflow waters. Using geochemical analyses of foraminiferal shells and grain size–specific detrital fractions, we show that erosion and chemical weathering of ophiolitic rocks markedly increased after 4 million years (Ma), coincident with widespread island emergence and gradual strengthening of Pacific zonal sea-surface temperature gradients. Together with supportive evidence for enhanced mafic weathering at that time from re-analysis of the seawater ^87^Sr/^86^Sr curve, this finding suggests that island uplift and hydroclimate change in the western Pacific contributed to maintaining high atmospheric CO_2_ consumption throughout the late Neogene.

## Teaser

Uplift of Indonesian islands since 4 Ma drove enhanced weathering of ophiolites and contributed to global climate cooling.

## INTRODUCTION

Arc-continent collisions are typically associated with emplacement of oceanic-derived mafic and ultramafic rocks at the Earth’s surface (*1*). When exposed on emerged lands, these highly weatherable rocks known as ophiolites are subject to erosion and their dissolution, coupled with alkalinity release and subsequent carbonate precipitation in the ocean, acts as a major sink for atmospheric carbon dioxide over million-year long time scales (*2*, *3*). This process is particularly enhanced whenever mafic and ultramafic rock exposure occurs in high-elevation tropical regions under warm and very wet conditions, where CO_2_ consumption by chemical weathering is most significant (*4*). Evidence for covariation between major glaciations and the distribution of low-latitude arc-continent collisions during the Phanerozoic has provided support for their role in driving global climate change over geological time scales (*2*, *3*, *5*). At present, active arc-continent collision occurs in tropical Southeast (SE) Asia, where the impingement of the Australian plate on the Banda-Sunda arc system has resulted in the obduction of massive, several kilometers thick, ophiolite complexes ultimately emplaced on top of many Indonesian islands such as Sulawesi, Timor, Seram, Halmahera, and New Guinea (*6*, *7*). In this region, the combination of high topography and monsoon rainfall sustains high erosion rates, accounting for about one third of the total sediment discharge exported to the ocean annually (*8*). Because of its presumed global impact on atmospheric CO_2_ consumption, via alteration of exposed ophiolitic rocks, chemical weathering in tropical SE Asia possibly played a major role in the evolution of late Cenozoic climate and notably in the onset of Northern Hemisphere glaciations (*9*, *10*). To date, however, this hypothesis still remains much debated, in particular due to inconsistencies between the presumed role of mafic weathering during the late Neogene cooling and the evolution of past seawater chemistry (*11*, *12*). In addition, major tectonic reorganization in SE Asia over the last few million years (*13*) has also modified regional ocean circulation patterns. After 4 to 3 million years (Ma), the constriction of the Indonesian seaway progressively reduced the inflow of warm and low-salinity tropical Pacific waters into the Indian Ocean, i.e., the so-called Indonesian Throughflow (ITF), possibly affecting climate at both regional and global scales, via atmospheric and oceanographic teleconnections (*14*–*16*). Nonetheless, to date, the cause-and-effect relationships between tectonics, climate, and the long-term evolution of erosion and mafic weathering in SE Asia remain elusive. Here, we present a ~8-Ma record of sediment provenance and seawater chemistry based on the application of neodymium (Nd) isotope and trace-element geochemistry to grain size–specific detrital fractions and foraminiferal shells. Our findings provide additional insight into feedbacks linking erosion, mafic weathering, climate, and island uplift in low-latitude arc-continent collision zones.

**Fig. 1.**
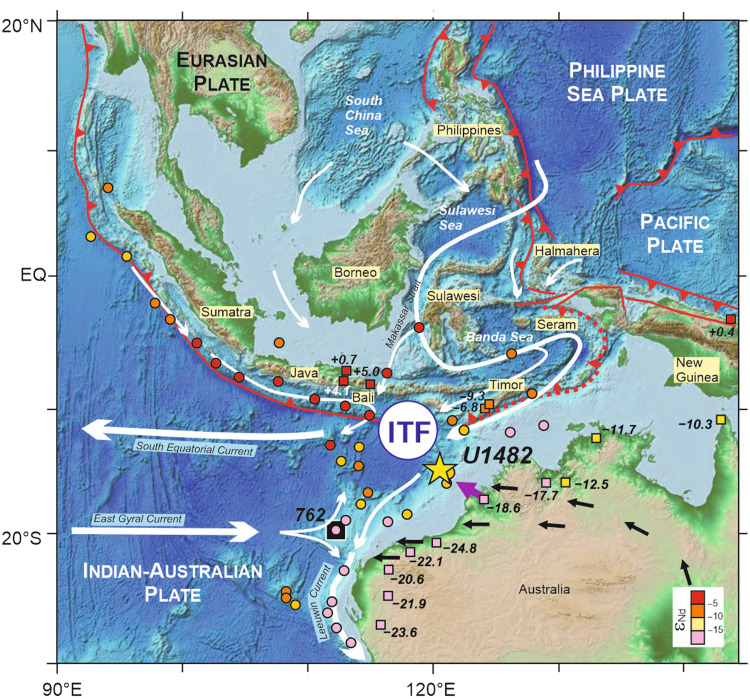
SE Asia topography and location of studied IODP Site U1482 at the NW Australian margin. Major plate tectonic boundaries in SE Asia are indicated in red lines (*7*), with the location of ongoing arc-continent collision along the northern Australian continental margin (red dotted line). The simplified flow pattern of today’s ocean surface currents (white arrows) (*20*), predominant wind directions in Australia (black arrows) (*68*) and local riverine discharge (purple arrow) near the core site illustrate the main transport mechanisms for the delivery of fine-grained detrital sediment to Site U1482. The distribution of Nd isotope ratios (ε_Nd_) in clay-size fraction of marine sediment surface samples (colored circles) (*21*) and river sediments (colored squares; table S6) (*69*) indicates that the ITF transports juvenile mafic-ultramafic sediment derived from SE Asia into the eastern Indian Ocean. The locations of IODP Site U1482 (yellow star) and ODP Site 762 (black square) (*27*) are also shown.

A suite of clay-rich nannofossil ooze samples (*n* = 46) was investigated from the upper ~370-mbsf (meter below seafloor) section of International Ocean Drilling Program (IODP) Site U1482 (15°3.32′S, 120°26.10′E, 1466 m water depth) drilled on the northwest (NW) Australian margin (*17*), at a location influenced by the ITF outflow (Fig. 1). To disentangle the effects of multiple provenances, Nd isotope ratios and trace-element abundances were determined in separate grain size fractions of the detritus (see Materials and Methods). Neodymium isotope ratios (^143^Nd/^144^Nd, or ε_Nd_) are not decoupled during sediment transport and can thus serve as powerful proxies for provenance (*18*). In addition, we used the nickel/thorium ratio to distinguish between mafic, ultramafic, and felsic material. In fine-grained sedimentary rocks, enrichments in detrital Ni generally relate to the presence of smectite formed via alteration of mafic or ultramafic rocks, while Th is mostly derived from felsic crustal rocks (*19*). In this study, the ITF advection of fine-grained mafic-ultramafic material carried from the Indonesian seas was traced using fine clays (<0.8 μm). Fine smectite-rich clays transported by the ITF, and ultimately deposited along the NW Australian margin (*20*), are associated with radiogenic Nd isotope composition (with high ε_Nd_ value), typical of juvenile source areas in tropical SE Asia (Fig. 1) (*21*). In contrast, the medium-coarse silt-size fraction (~10 to 63 μm; which corresponds to the noncohesive, sortable, detrital size-fraction of the sediment) was analyzed to reconstruct the export of unradiogenic (with low ε_Nd_) felsic crustal material from nearby Australian source regions (Fig. 1). Complementary geochemical analyses were also performed on the fine, cohesive, silt fraction (~4 to 12 μm), which can be influenced by both aeolian and ocean current transport. Our approach also included Nd isotope analyses of bulk foraminiferal separates to constrain the long-term evolution of seawater chemistry (see Materials and Methods). In marine sediment records, Nd and other rare earth elements (REE) in foraminifers are hosted by Fe-Mn oxyhydroxide coatings formed after deposition at the sediment-seawater interface, hence acquiring the Nd isotopic composition of bottom waters (*22*).

## RESULTS

**Fig. 2.**
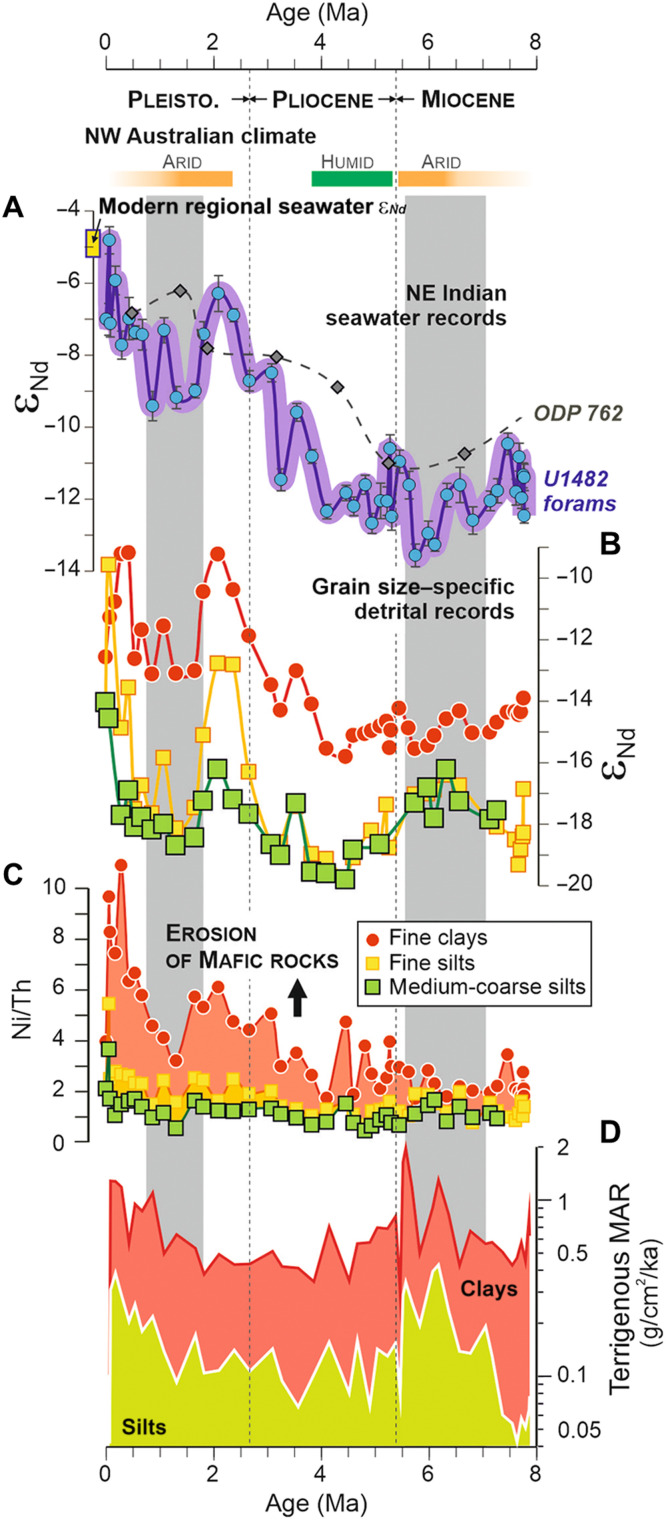
Geochemical proxy records of the evolution of sediment provenance at Site U1482 over the last 8 Ma. (**A**) ε_Nd_ values in bulk foraminiferal separates, as a proxy for dissolved/particulate exchange processes in seawater. A seawater ε_Nd_ record derived from the nearby site ODP762 (gray dotted line, see location in Fig. 1) (*27*) and the range of modern seawater ε_Nd_ values at the NW Australian margin (yellow box) (*25*) are shown for comparison. (**B**) ε_Nd_ values in fine clay (<0.8 μm), fine silt (4 to 12 μm), and medium-coarse silt (10 to 63 μm) fractions of the detrital sediment. The pronounced ε_Nd_ shift identified after ~4 Ma in foraminiferal shells, fine clay, and fine silt fractions indicate enhanced contribution of juvenile mafic-ultramafic material transported by the ITF. (**C**) Ni/Th ratios in fine clay, fine silt, and medium-coarse silt fractions at Site 1482, as a provenance tracer for mafic-ultramafic (ophiolitic) versus felsic crustal material. (**D**) Terrigenous mass accumulation rates for clay- and silt-size fractions at Site U1482. Vertical gray bands refer to periods of inferred strong dust activity, consistent with broad paleoclimate reconstruction in NW Australia (see yellow and green boxes) (*23*, *24*).

Except for the most recent Quaternary period (<~0.2 Ma, which falls outside the scope of this study), medium-coarse silt fractions (~10 to 63 μm) at Site U1482 display a range of unradiogenic ε_Nd_ composition between −19.8 and −16.2 (Fig. 2 and table S1) and constantly low Ni/Th ratios (mean value 1.1 ± 0.4; 1 SD; *n* = 44; Fig. 2C and table S4), which indicate felsic sediment contributions from nearby Australia. The lowest ε_Nd_ values for medium-coarse silts occur between ~5.2 and 3.8 Ma (with a mean ε_Nd_ −19.3 ± 0.5; *n* = 5), at a time coinciding with prevailing humid conditions in NW Australia (Fig. 2) (*23*, *24*). Because river sediments from NW Australia exhibit similar ε_Nd_ composition (Fig. 1 and table S6), this observation confirms that proximal riverine inputs influenced detrital sedimentation at Site U1482 during this wet interval (*23*). The late Miocene and Pleistocene periods between ~7.2 to 5.8 Ma and ~2.1 to 0.3 Ma, respectively, during which arid conditions prevailed in NW Australia, are associated with coarser silt fractions having slightly more radiogenic ε_Nd_ composition (−17.5 ± 0.7; *n* = 17), indicating contributions from remote dust source regions in Australia (Fig. 1). These two time intervals also coincide with periods of higher terrigenous accumulation rates at Site U1482 (Fig. 2D), which confirm that aeolian activity strongly influenced detrital sedimentation at the NW Australian margin over the last 8 Ma, except between 5.3 and 3.8 Ma and the late Pleistocene (*25*). Overall, the unradiogenic ε_Nd_ composition of studied medium-coarse silts clearly point toward the near absence at Site U1482 of “sortable” (>10 μm) detrital particles transported from the Indonesian seas, possibly implying relatively low current speeds of the ITF for most of the time interval considered in this study.

At Site U1482, provenance proxy records for both fine silt (~4 to 12 μm) and medium-coarse silt (~10 to 63 μm) fractions display similar trends until ~4.1 Ma (Fig. 2). From that time onward, the Ni/Th ratio (and to a lesser extent ε_Nd_) shifts toward slightly higher values (2.3 ± 0.9; *n* = 21; Fig. 2). Similarly, fine clays (<0.8 μm) show relatively low ε_Nd_ (−14.8 ± 0.5; *n* = 23) and Ni/Th (1.5 ± 0.8; *n* = 25) values until ~4.1 Ma, indicative of dominant felsic sediment inputs from Australia, before gradually shifting toward more radiogenic ε_Nd_ (up to −9.2) and much higher Ni/Th (up to 10.9) compositions. Compared to the coarser noncohesive silt fraction, both clays and fine silt detrital fractions are more efficiently transported by ocean surface currents. Therefore, the abovementioned provenance changes starting from about 4 Ma are best interpreted as reflecting a gradual increase in the ITF export of fine-grained mafic-ultramafic ophiolitic material from the Indonesian Archipelago.

Bulk foraminiferal separates at Site U1482 display similar long-term ε_Nd_ evolution over the past 8 Ma, also indicating a pronounced shift after ~4 Ma toward modern regional seawater ε_Nd_ values (between −5.2 and −4.5; Fig. 2A) (*26*). This evolution is consistent with another regional record of bottom-water chemistry based on biogenic carbonate analyses (*27*). At present, the marine Nd budget at the NW Australian margin is dominated by upstream interactions between seawater and marine sediments deposited at the margins of Indonesian seas (*26*). At active margins, early diagenetic processes typically proceed with the dissolution of reactive mafic minerals in organic-rich sediments under anoxic conditions (*28*, *29*). This process is presumably accompanied by large benthic fluxes from marine sediments, which can shift the Nd isotope composition of overlying bottom waters toward radiogenic values (*30*). At Site U1482, geochemical data for interstitial fluids indicate limited early diagenesis in subsurface sediments and the absence of marine silicate weathering (*17*). However, sedimentary records retrieved from the northern margin of New Guinea (IODP U1484, U1485) display high pore-water alkalinity levels (up to ~50 μM), which provide clear evidence for ongoing dissolution of reactive silicate minerals in the upper sedimentary column (*17*). In addition to marine silicate weathering, partial dissolution of volcanogenic particles exported to the ocean can also release substantial amounts of dissolved radiogenic Nd in seawater (*31*). On this basis, we interpret the observed ε_Nd_ foraminiferal shift toward more radiogenic signatures after 4 Ma as reflecting intensifying sediment-seawater exchange processes in the Indonesian seas, due to enhanced dissolution of reactive mafic minerals in both the water column and anoxic marine sediments, followed by subsequent export of more radiogenic waters via the ITF. Superimposed onto this general trend, we also note that the periods of arid conditions and inferred strong dust activity at ~7.2 to 5.8 Ma and ~2.1 to 0.3 Ma were accompanied by corresponding ε_Nd_ shifts in bulk foraminiferal separates (Fig. 2), thereby suggesting that the dissolution of windblown particles in the water column during periods of enhanced aeolian dust inputs may have also influenced the long-term foraminiferal ε_Nd_ variability.

To summarize, our findings based on separate grain size detrital fractions and foraminiferal assemblages indicate that both mafic material transport and sediment-seawater exchange processes gradually increased in the Indonesian seas from the Early Pliocene.

## DISCUSSION

### Early Pliocene intensification of mafic sediment transport by the ITF linked to the emergence of the Maritime Continent

**Fig. 3.**
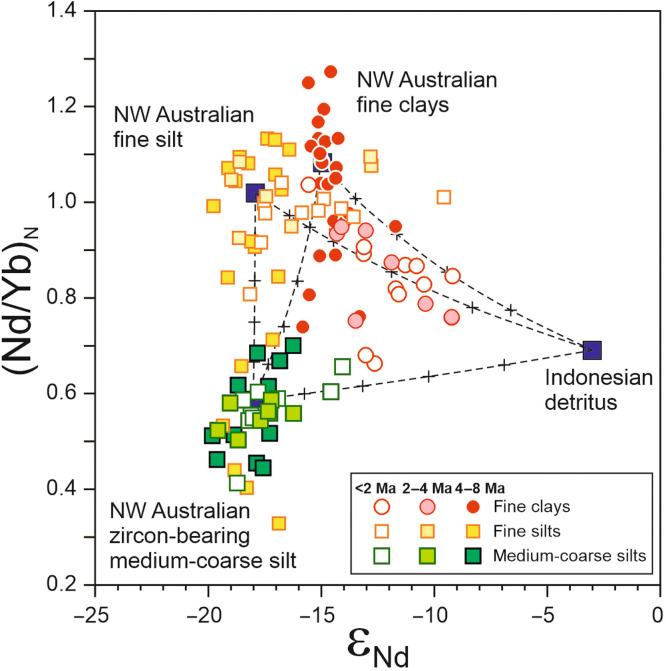
Quantitative constraints on late Neogene provenance changes at Site U1482. Plot of Nd isotopes (ε_Nd_) versus shale-normalized neodymium/ytterbium ratios (Nd/Yb)_N_ for U1482 detrital fractions, with inferred average end-member compositions for NW Australian fine clays (<0.8 μm), zircon-depleted fine silts (4 to 12 μm), zircon-bearing medium-coarse silts (10 to 63 μm), and detritus corresponding to the mafic material eroded from Indonesian islands. Mixing lines (dotted lines) between different end-members are shown with black crosses indicating tick marks at increments of 20%. Nd isotopic compositions and (Nd/Yb)_N_ ratios for end-members are listed in table S7.

Quantitative constraints on past provenance changes can be obtained using mixing models combining both ε_Nd_ and (Nd/Yb)_N_ end-member compositions for size-specific Australian and Indonesian sediment sources (see table S7). While total terrigenous mass accumulation rates at Site U1482 remained near-constant between ~4 and 2 Ma (Fig. 2D), the relative contribution of mafic-ultramafic material to the fine clay fraction increased up to 60 to 70% during this time interval (Fig. 3), hence clearly indicating enhanced mafic sediment fluxes from tropical SE Asia. The observed shifts in fine clay records at Site U1482 could result either from an increase in strength of the ITF or, alternatively, from higher concentrations of Indonesian suspended sediment in the water column. The first hypothesis seems unlikely because the progressive constriction of the Indonesian seaway, while being accompanied by a switch in the source of waters feeding the ITF at that time, from Southern to Northern Pacific subtropical waters, most likely resulted in an overall weakening of the ITF outflow (*13*–*16*, *32*). In addition, while high-latitude cooling may have possibly promoted intensification of the overturning circulation and an increase in ITF strength after 4 Ma, which would have contributed to the observed Nd isotope shifts in both fine clays and foraminifers, this hypothesis is not supported by Nd isotope records from nearby sediment cores, because those records indicate very little ε_Nd_ glacial-interglacial variability in the clay fraction during the Pleistocene (*21*, *33*). By inference, at Site U1482, the observed shifts indicating enhanced ITF transport of mafic-ultramafic material are probably best explained by a change in the erosional flux to the Indonesian seas after the Early Pliocene.

In tropical SE Asia, the area of subaerially exposed land has increased sporadically since the mid-Miocene, in response to arc-continent collision between Australia and the Sunda-Banda arc system (*3*, *9*, *34* and references therein). Nevertheless, regional syntheses of late Neogene tectonic activity indicate that the Early Pliocene coincided with accelerated surface uplift and widespread emergence of Indonesian islands (*9*, *34*, *35*). New Guinea, for instance, was largely submerged until the Early Pliocene, and its rapid emergence due to major mountain building occurred in the last 5 Ma (*36*). Timor and most of the islands of the Outer Banda arc also emerged over the last few million years (*37*, *38*). More specifically, paleotopographic reconstructions in West Timor indicate that the emergence of Timor above sea level at ca. 4.5 Ma was followed by a period of markedly increasing uplift rates (up to 5 mm/year) between 3.1 and 2.2 Ma (*38*), hence coinciding with the timing of the radiogenic ε_Nd_ shifts recorded by fine clays and bulk foraminifers at Site U1482 (Fig. 2).

During the late Neogene period of accelerated uplift, increasing land area and higher topography, i.e., the two main parameters controlling sediment yields in river systems (*8*), probably drove enhanced erosional inputs into the Indonesian seas. At that time, the accretion of the Banda Terrane after ~4 Ma, as a result of the collision between the Banda volcanic arc and the northern continental margin of Australia, uplifted and exposed extensive sections of forearc basement and sedimentary cover on Timor and other islands of the Outer Banda arc (*39*). Considering the close proximity of these islands to the study site, erosional inputs from the Banda Terrane to the foreland basin system developed on the Australian margin (*39*) probably strongly influenced the detrital sedimentation at Site U1482 after 4 Ma. Nevertheless, clay-size siliclastic fractions in marine sediments are likely to integrate source contributions at a larger regional scale. A detailed multi-isotopic (Sr, Nd, and Pb) provenance investigation indicates that clays deposited at present along the NW Australian margin are probably derived from the entire region draining the Banda and Timor seas (Fig. 1) (*21*). Since other high mountains also emerged contemporaneously on the margins of the Banda Sea, such as in Buru, Seram, Halmahera, and Sulawesi, one of the largest islands in SE Asia (*34*, *38*, *40*), our provenance proxy records at Site U1482 can probably be linked to the regional phase of accelerated surface uplift across the Indonesian seas during the Pliocene.

Considering that the total land area of islands in SE Asia has more than doubled in size over the last 5 Ma (*9*, *10*), we therefore posit that the long-term increase in mafic material transport inferred from our proxy records for fine clays was mostly caused by increasing riverine fluxes of sediment loads, following the regional emergence of the so-called Maritime Continent (*9*).

### Interactions between erosion, uplift, and hydroclimate in the western tropical Pacific over the last 4 Ma

Widespread evidence exists for the co-evolution of erosion and uplift in tropical SE Asia since the late Neogene (*7*, *34*, *35*, *37*, *38*, *41*–*43*). Accelerated uplift and island emergence were generally accompanied by massive deposition of marine siliciclastic turbidites in subsiding proximal sedimentary basins, indicative of high erosion rates and strong vertical motions (*34*, *37*, *38*, *41*–*43*). For instance, in Borneo, the largest land mass in the area, which was already almost fully emerged 5 Ma ago (*9*), huge volumes of sediment corresponding to an average thickness of eroded crust of ~5 to 6 km have been transferred to nearby deep marine basins since the Miocene (*43*–*45*). Rapidly subsiding sedimentary basins offshore fast-eroding mountainous islands are thought to have caused deep crustal movements that flowed laterally into areas already elevated, hence driving further uplift [see figure 21 in (*45*)]. Such deep crustal flow possibly contributed to maintaining high exhumation rates and erosional inputs that drove further subsidence in adjacent offshore basins (*45*). Such mechanism may have contributed to the formation of high mountains on SE Asian islands in the last 5 Ma (*43*, *45*). In addition to sedimentary fluxes, the sum of dissolved solids released by chemical weathering of highly erodible ophiolitic rocks and ultimately exported by rivers also probably accounted for a substantial fraction of the total denudation rate and unloading of emerged islands, in the order of ~10% based on present-day estimates (*8*).

**Fig. 4.**
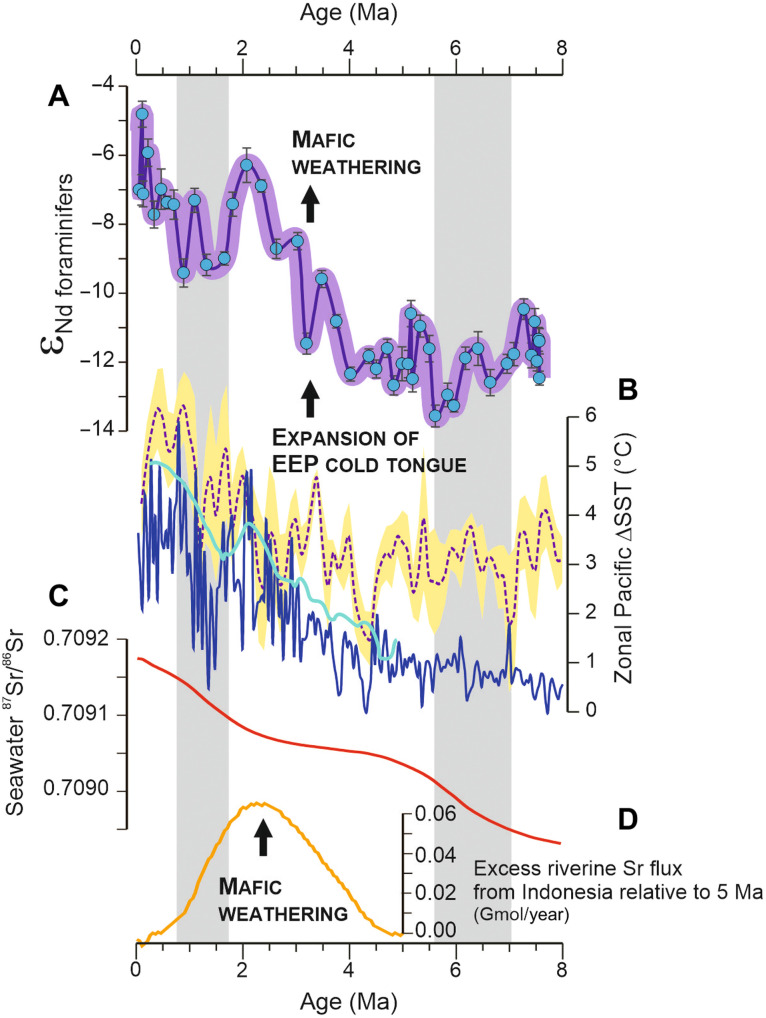
Evolution of mafic weathering in the Indonesian region in the context of hydroclimate change over the last 8 Ma. (**A**) ε_Nd_ values in foraminiferal shells at Site U1482, as a proxy for bottom-water chemistry reflecting sediment-seawater exchange processes. (**B**) Pacific zonal (west-to-east) sea-surface temperature gradients (ΔSST) as proxies for the intensity of Walker Circulation and the expansion of the eastern equatorial Pacific (EEP) cold tongue. The dark blue line represents the west-to-east SST difference between IODP Site U1337 (4°N, 123°W) and ODP Site 846 (3°S, 91°W) in the eastern EEP (*48*). The light blue line (*47*) and purple dotted line (*49*) represent computed changes of zonal SST gradients between the Western Pacific Warm Pool (WPWP) and the EEP. Yellow shading represents the associated uncertainty (1 SE) on zonal SST gradient (*49*). (**C**) Late Neogene evolution of the Sr isotope composition of seawater (*59*). (**D**) Excess riverine Sr fluxes from Indonesian islands (Gmol/year; relative to 5 Ma) required to account for the observed seawater ^87^Sr/^86^Sr plateau between ~4.5 and 2 Ma. Gray vertical bands refer to periods of inferred strong dust activity (*23*, *24*).

Our provenance proxy data also suggest a link with the long-term evolution of tropical Pacific climate (*46*–*49*), more specifically with the gradual strengthening of the zonal (west-to-east) sea surface temperature (SST) difference during the Pliocene (Fig. 4B). While reduced zonal SST temperature gradients prevailed in the tropical Pacific between ~4 and 8 Ma (Fig. 4B), the expansion of the Pacific “cold tongue” after ~4 Ma—a region of relatively cool water in the eastern equatorial Pacific (EEP)—was accompanied by a strengthening of the Walker Circulation (*46*–*48*). To date, there is only limited understanding of the behavior of the Western Pacific Warm Pool (WPWP) and the repercussions of the late Neogene expansion of the EEP cold tongue on regional monsoons. While extra-tropical forcing also affects the WPWP state and regional rainfall patterns (*47*), previous work suggested that the progressive intensification of the Walker atmospheric cell inferred from paleo-SST records may have resulted in moisture redistribution across the EEP at that time, leading to increasing regional convection and wetter conditions in the western tropical Pacific after ~4.5 to 4.0 Ma (*9*, *50*, *51*). An increase in chemical weathering patterns recorded in a drilled sedimentary record at the northern margin of New Guinea has been recently interpreted as reflecting enhanced rainfall following the expansion of the EEP cold tongue and associated strengthening of the Walker Circulation (*52*). Similarly, at Site U1482, the evidence for reduced export of eroded mafic-ultramafic material from the Indonesian seas inferred from our proxy records between ~1.8 and 0.8 Ma (Figs. 2 and 4), when weaker zonal temperature gradients (*47*, *48*) possibly resulted in dryer conditions in tropical SE Asia and enhanced aeolian activity in NW Australia, also points toward a putative link between Pacific SST gradients and WPWP hydroclimate.

On this basis, after ~4 Ma, in a context of active arc-continent collision and associated tectonic shortening, a regional hydroclimate shift toward enhanced rainfall in the western tropical Pacific may have driven synchronous acceleration of erosion and riverine discharge on emerged islands (*52*). Such climatically induced increase in erosional unloading combined with concomitant sedimentary loading in nearby marine basins could have triggered rapid isostatic compensation (*53*), possibly leading to higher exhumation rates across the Maritime Continent (*45*).

Future work would be required to provide more direct evidence for enhanced rainfall in the western Pacific after 4 Ma and to quantitatively assess the extent to which the potential climate-driven acceleration of erosion may have contributed to isostatic uplift at that time. Nevertheless, considering the high density (up to ~3.3 g/cm^3^), thickness (several kilometers thick), and widespread occurrence of ophiolitic peridotites across SE Asian islands (e.g., Timor, Seram, Sulawesi, New Guinea, Borneo, and Philippines) (*6*), it is very likely that the erosion of these highly erodible ultramafic rocks will have contributed to a strong isostatic response and surface uplift, possibly playing a key role in the accelerated emergence of high-standing islands in tropical SE Asia after 4 Ma.

### Implications for the global impact of ophiolite weathering and links with late Neogene cooling

One important implication of our results is that the degree of atmospheric CO_2_ drawdown associated with chemical weathering of exhumed ophiolitic rocks on emerging islands probably scaled positively with the growth of the Maritime Continent and the possible concomitant intensification of rainfall after ~4 Ma. In principle, a decrease in atmospheric CO_2_ concentrations should imply a weaker silicate weathering feedback strength, resulting overall in lower chemical weathering fluxes (*54*). However, in the context of uplifting islands and a regional hydroclimate shift toward probably wetter conditions, the late Neogene decline in atmospheric CO_2_ would instead have caused an increase in land surface reactivity and efficiency of weathering, hence resulting in higher chemical weathering fluxes.

As mentioned earlier, a global impact of mafic weathering on late Neogene climate change is generally not supported by proxy records for the long-term evolution of seawater chemistry (*12*, *55*, *56*). For instance, while sustained chemical weathering of ophiolites would be expected to have driven the Sr isotope composition of seawater toward unradiogenic values, the long-term marine ^87^Sr/^86^Sr curve evolved instead in opposite direction during the Neogene; a feature that has long been interpreted as reflecting enhanced discharges from Himalayan rivers following the uplift of the Tibetan Plateau (*55*) or, more recently, an increase in the global efficiency of weathering (*54*). Nevertheless, dissolved fluxes of radiogenic Sr from Himalayan rivers (*57*) and other high-elevation catchments worldwide (*58*) are strongly influenced by the dissolution of carbonaceous rocks, with limited effects on the long-term *p*CO_2_, hence suggesting that the marine Sr cycle may have been partly decoupled from silicate weathering fluxes during the Cenozoic. In addition, the late Neogene Sr isotope seawater curve indicates a break in slope between ~4.2 and 2.2 Ma (Fig. 4C), corresponding to a ~5-fold decrease relative to both preceding and subsequent dryer periods in tropical SE Asia (Fig. 2), which were characterized by a steeper ^87^Sr/^86^Sr increase at an average rate of ~50 × 10^−6^/Ma (*59*, *60*). The timing of the observed ^87^Sr/^86^Sr “plateau” on the marine Sr isotope curve coincides with the period of high mafic weathering inferred from our proxy records (Fig. 4A), thereby suggesting that unradiogenic Sr inputs from SE Asia possibly influenced the global marine Sr cycle at that time. To test this hypothesis, a numerical approach was used for estimating the riverine Sr flux from Indonesian islands required to explain the observed seawater ^87^Sr/^86^Sr plateau between ~4.2 and 2.2 Ma. This model considers global Sr fluxes and isotopic ratios for the contributions of silicate and carbonate rocks to riverine inputs and interactions between oceanic crust and seawater at mid-ocean ridges (see Materials and Methods). Geochemical and Sr isotopic ratios for Indonesian mafic rocks used for calculation were inferred from precompiled rock compositions for Sulawesi, Halmahera, and other Banda islands, yielding an average value of 0.7085 (± 0.0069 1 SD, *n* = 636; table S8). The model output indicates that the abovementioned plateau in the seawater ^87^Sr/^86^Sr curve is consistent with a gradual increase in mafic weathering between ~4.5 and 2 Ma (Fig. 4D), hence in agreement with our provenance proxy data at Site U1482. After ~2 Ma, the observed steady increase of the marine Sr isotope curve could reflect reduced rates of mafic weathering in Indonesia or, alternatively, a change in the Sr isotope composition of riverine inputs from the Himalayan-Tibetan Plateau toward more radiogenic values (*60*). While clearly suggesting that accelerating chemical weathering of Indonesian ophiolites most likely influenced the Sr isotope composition of seawater over the last 5 Ma, the corresponding excess in mafic Sr fluxes (up to 0.06 Gmol/year between ~3 and 2 Ma, relative to the initial model input values at 5 Ma; Fig. 4D) remains small when compared to the estimated present-day global riverine Sr flux to the oceans (~47.6 Gmol/year) (*61*).

Marine silicate weathering could possibly help reconciling the apparent inconsistency between the presumed impact of mafic weathering in late Neogene global cooling and the marine strontium budget. In addition to subaerial ophiolite weathering, our foraminiferal Nd isotope record indeed suggests that enhanced mafic sediment delivery to the Indonesian seas during the Pliocene was followed by increasing silicate weathering in marine sediments. In anoxic sediments, such as those encountered at the northern New Guinean margin (*17*), the dissolution of reactive silicate minerals releases substantial amounts of major cations and alkalinity, which drive precipitation of authigenic carbonates and therefore act as a net carbon sink (*28*, *29*). While accelerated submarine silicate weathering following late Neogene erosion of SE Asian islands most likely represented an additional sink for atmospheric CO_2_, probably as large as the CO_2_ consumption rate associated with subaerial silicate weathering (*28*), the corresponding benthic flux of Sr and other elements to seawater would have been quantitatively buffered by authigenic carbonate precipitation in marine sediments (*62*). Future observational and modeling studies would be required to further assess whether submarine silicate weathering can reconcile the apparent conflicts in the interpretation of late Cenozoic marine geochemical cycles in the context of extensive ophiolite weathering. Nevertheless, our main finding—linking the emergence of Indonesian islands with enhanced mafic weathering in a context of regional hydroclimate change—provides direct support to the hypothesis that arc-continent collision in tropical SE Asia, together with other important tectonic forcings at that time (*63*, *64*), may have been instrumental in driving global climate cooling during the late Neogene.

## MATERIALS AND METHODS

### Age model and mass accumulation rates

The age-depth model for Site U1482 was established using onboard biostratigraphic correlation based on calcareous nannofossils and planktonic foraminifers (*17*). The age model was obtained with a fourth-order polynomial regression including all biostratigraphic datum levels (*17*). Average sedimentation rates are ~5.9 cm/ka during the late Miocene, ~3.3 cm/ka in the early Pliocene, and ~7 cm/ka in the Pleistocene. Note that there is evidence for a possible short hiatus at ~300 mbsf, which spans a series of planktonic foraminifer biohorizons from 6.08 to 6.60 Ma, and for minor sediment reworking during the lower Pleistocene (~0.6 to 1.5 Ma) interval (*17*). However, considering the relatively low temporal resolution in this study, these artifacts have negligible influence on data interpretation. Mass accumulation rates for detrital clay- and silt-size fractions were calculated using onboard dry density data (g/cm^3^), linear sedimentation rates (cm/ka) and relative proportions of fine clay (<0.8 μm), clay (0.4 to 2 μm), fine silt (4 to 12 μm), and medium-coarse silt (10 to 63 μm) (table S1).

### Sample preparation and grain size separation

Site U1482 samples were first sieved at 125 and 63 μm to isolate bulk foraminiferal (>125 μm) and fine-grained sediment (<63 μm) fractions, respectively. A sequential leaching procedure was applied to fine-grained sediment fractions to successively remove carbonate, iron oxide, and organic compounds (*65*). Between ~3 and 4 g of dried bulk powder was placed into 50-ml polyethylene centrifuge tubes with 20 ml of 5% (v/v) acetic acid (AcOH) and left for 3 hours on mechanical shaker to remove carbonate phases. The second leaching step involved addition of 20 ml of mixed 20% (v/v) AcOH–0.5 M hydroxylamine hydrochloride solution to achieve quantitative extraction of more resistant carbonate phases and Fe-Mn oxides (2 days on mechanical shaker). The resulting residual fractions were treated with 20 ml of 35% (v/v) hydrogen peroxide (H_2_O_2_) for removal of organic compounds (2 days on mechanical shaker). The medium-coarse silt (10 to 63 μm), fine silt (4 to 12 μm), and the finest clay-size (<0.8 μm) fractions of the detrital residues were then separated by differential centrifugation (see the Supplementary Materials). In addition, a suite of clay-size detrital fractions separated from various river sediment samples (*n* = 10) in NW Australia and SE Asia was analyzed Nd isotopes, following the protocol described in (*18*).

### Nd isotope and trace element analyses

For geochemical analyses, between ~15 and 40 mg of powdered sediment of grain size detrital fractions was digested in polytetrafluoroethylene (PTFE) vials with concentrated ultrapure HF-HNO_3_-HCl (7 days on hotplate at 140°C). Bulk foraminiferal separates were dissolved gently by dropwise addition of ultrapure 1 M AcOH, following the procedure described in (*22*).

Neodymium was separated by conventional ion chromatography, and Nd isotopic compositions were determined with a Thermo Fisher Scientific Neptune multicollector inductively coupled plasma mass spectrometry (ICP-MS) at the Pôle Spectrométrie Océan (Brest, France) using sample-standard bracketing methods. Mass bias corrections were made using the exponential law, using ^146^Nd/^144^Nd = 0.7219. During the course of this study, repeated analyses of JNdi-1 standard solutions gave ^143^Nd/^144^Nd of 0.512114 ± 0.000006 (2σ, *n* = 25), in full agreement with recommended value of 0.512115. Associated uncertainties on JNdi-1 analyses correspond to an external reproducibility of ±0.12 ε (2σ). Epsilon values, i.e., ε_Nd_ = [(^143^Nd/^144^Nd)_measured_/ (^143^Nd/^144^Nd)_CHUR_ − 1] × 10^4^, were calculated using the present-day chondritic (CHUR) value for ^143^Nd/^144^Nd (0.512630) (*66*).

Trace element abundances for both grain size detrital fractions and bulk foramineral separates were determined at the Pôle Spectrométrie Océan (Brest, France) with a Thermo Fisher Scientific Element XR sector field ICP-MS. The in-run uncertainties on all measurements were better than 2%. The precision and accuracy of our data were assessed by analyzing a series of certified reference materials for silicate (AGV-1, DR-N, and WS-E) and carbonate (JLs-1, CAL-S) rocks. The precision of measurements given as relative SD was generally <5% for REE and other trace elements (Rb, Sr, Zr, Y, Ba, Hf, Th, U, Ni, and Co). All results obtained for reference materials were in agreement (<10%) with reference values from the literature.

### Simplified mass balance model for the seawater Sr isotope composition

The progressive rise of seawater ^87^Sr/^86^Sr during the Neogene has been linked to enhanced continental weathering fluxes of radiogenic Sr and/or changes in the ^87^Sr/^86^Sr ratio of source rocks exposed in the uplifting Himalayan-Tibetan Plateau [e.g., (*55*, *57*, *60*)]. In this study, we first detrended the evolution of seawater ^87^Sr/^86^Sr over the last 5 Ma (*59*). Once linearly detrended, the marine ^87^Sr/^86^Sr curve displays a gradual decrease between 5 and ~2.5 Ma, from ~0.709035 to 0.709000 (fig. S4). A simple numerical model of the Sr isotope mass balance in the ocean was used to investigate whether this decrease could reflect enhanced riverine Sr fluxes derived from mafic weathering in tropical SE Asia.

At 5 Ma, the mass balance equation for the Sr marine cycle can be written as follows

$$F_{\mathrm{sw}}^{0}\;\cdot (r_{\mathrm{sw}}-r_{\mathrm{ocean}})+F_{\mathrm{cw}}\;\cdot (r_{\mathrm{cw}}-r_{\mathrm{ocean}})+F_{\mathrm{MOR}}\;\cdot (r_{\mathrm{MOR}}-r_{\mathrm{ocean}})=0$$ (1)

where $F_{\mathrm{sw}}^{0}$ is the global flux of Sr released to the ocean by continental silicate weathering before 5 Ma. *F*_cw_ and *F*_MOR_ correspond to Sr fluxes associated with continental carbonate weathering and oceanic crust-seawater interactions at mid-ocean ridges, respectively. *r*_ocean_ is the seawater ^87^Sr/^86^Sr, while *r*_sw_, *r*_cw_, and *r*_MOR_ represent the mean ^87^Sr/^86^Sr values for continental chemical weathering of silicate and carbonate rocks (table S8).

The second key equation of the model is based on the classical silicate weathering feedback (*67*), whereby the CO_2_ consumption by global silicate weathering must be very close to the solid Earth total CO_2_ degassing *F*_degas_, due to the climate dependency of continental weathering. Translated into Sr fluxes, this constraint can be written as

$$\alpha_{\mathrm{ophio}}\;\cdot \;F_{\mathrm{ophio}}+\alpha_{\mathrm{sw}}\;\cdot \;F_{\mathrm{sw}}=\alpha_{\mathrm{MOR}}\;\cdot \;pF_{\mathrm{MOR}}$$ (2)

where *F*_ophio_ is the Sr flux from chemical weathering of ophiolitic rocks exposed on emerged SE Asian islands, and *F*_sw_ is the total Sr released from chemical weathering of other silicate rocks worldwide. The α factors correspond to the average (Ca + Mg)/Sr ratios of mafic Indonesian rocks, the upper continental crust and the oceanic crust. Given that *F*_degas_ is proportional to $F_{\mathrm{sw}}^{0}$ (silicate weathering feedback), and assuming a constant *F*_degas_ over the last 5 Ma, Eq. 2 becomes

$$\alpha_{\mathrm{ophio}}\;\cdot \;F_{\mathrm{ophio}}+\alpha_{\mathrm{sw}}\;\cdot \;F_{\mathrm{sw}}=\alpha_{\mathrm{sw}}\;\cdot \;F_{\mathrm{sw}}^{0}$$ (3)

and the marine Sr isotope budget after 5 Ma gives

$$F_{\mathrm{sw}}\;\cdot \;(r_{\mathrm{sw}}-r_{\mathrm{ocean}})+F_{\mathrm{ophio}}\;\cdot \;(r_{\mathrm{ophio}}-r_{\mathrm{ocean}})+F_{\mathrm{cw}}\;\cdot \;(r_{\mathrm{cw}}-r_{\mathrm{ocean}})+F_{\mathrm{MOR}}\;\cdot \;(r_{\mathrm{MOR}}-r_{\mathrm{ocean}})=0$$ (4)

Equations 1, 3, and 4 can be solved for $F_{\mathrm{sw}}^{0}$, *F*_sw_, and *F*_ophio_. *F*_cw_ is held constant at 6 Gmol Sr/year (any error on this flux is never critical, given that *r*_cw_ is always close to *r*_ocean_). The parameters used are given in table S8. In this simple model, we assume that the marine Sr budget always remained close to steady-state conditions over the last 5 Ma. While the steady-state assumption is most likely valid for the carbon cycle, it was certainly not the case for the marine Sr isotope cycle, considering its long residence time (>3 Ma) in seawater. In a more complex transient model, the amount of Sr derived from mafic weathering of Indonesian rocks required to produce the observed seawater ^87^Sr/^86^Sr plateau between ~4.5 and 2.5 Ma would have been presumably higher. Consequently, our simple model most likely provides a “lower bound” estimate of the contribution of mafic weathering in SE Asia to the Sr isotopic composition of seawater for the late Neogene.

## Supplementary Materials

### This PDF file includes:

Supplementary Text Figs. S1 to S4 Legends for tables S1 to S8 References
