# Supplementary material for: Accelerated mafic weathering in Southeast Asia linked to late Neogene cooling
Source: Sci Adv. 2023 Mar 29;9(13):eadf3141. doi: 10.1126/sciadv.adf3141 (PMC10058235; doi:10.1126/sciadv.adf3141)
Supplement: Supplementary file 1 — Supplementary Text Figs. S1 to S4 Legends for tables S1 to S8 References [file sciadv.adf3141_sm.pdf]

Science Advances
AAAS

Supplementary Materials for

Accelerated mafic weathering in Southeast Asia linked to late Neogene cooling

Germain Bayon *et al.*

Corresponding author: Germain Bayon, gbayon@ifremer.fr

Sci. Adv. **9**, eadf3141 (2023)
DOI: 10.1126/sciadv.adf3141

The PDF file includes:

Supplementary Text
Figs. S1 to S4
Legends for tables S1 to S8
References

Other Supplementary Material for this manuscript includes the following:

Tables S1 to S8

Supplementary Text

Grain-size separation procedure

Following our sequential leaching procedure, different grain-size fractions of U1482 detrital sediments were separated by differential centrifugation, using parameters (duration, rpm) defined with the freeware Sedicalc (*70*). First, 25 ml of ultrapure (MQ) H_2O were added to the 50 ml centrifuge tube (**T0**) containing the bulk detrital residue, shaken vigorously, and centrifuged for 2 min at 200 rpm. The 25-ml supernatant was transferred into a clean centrifuge tube (**T1**). Another 25 ml MQ-H_2O was added to the residue in **T0**, mixed thoroughly again, and centrifuged for another 2 min at 200 rpm. This latter supernatant was discarded and the remaining residue in **T0**, corresponding to the medium-coarse silt-size fraction (10-63 µm), was placed in an oven for drying. Tube **T1** was shaken thoroughly and centrifuged 2 min at 1000 rpm. The supernatant was transferred in a new tube **T2**, while the remaining residue in **T1**, corresponding to the fine silt (4-12 µm) fraction was rinsed in 25 ml before being dried. The same procedure was repeated at 2000 rpm, 3000 rpm and 4000 rpm, yielding residual fractions corresponding to coarse clays (1-4 µm ; stored in **T2**), clays (0.4-2 µm ; in **T3**) and fine clays (<0.8 µm ; in **T4**), respectively. Note that the final 25 ml MQ-H_2O rinse in **T3** was not discarded but transferred instead to **T4**. The validity of our protocol for grain-size separation was assessed using a suite of various river and marine sediment samples (n=8 ; Fig. S1), including one sample from Site U1482 (U1482A_1H1_145/150 cm). Following differential centrifugation, particle-size analyses of test samples were performed using a Mastersizer 3,000 laser diffraction particle size analyzer. The general good agreement observed for particle size distribution measurements in all test samples demonstrates the reproducibility of our separation procedure (Fig. S1). In this study, three separate grain-size fractions were analysed for provenance studies : coarse-medium silts (10-63 µm), fine silts (4-12 µm), and fine clays (<0.8 µm).

Preservation of bottom-water Nd isotopic signatures in bulk foraminiferal separates

Rare earth element (REE) abundances were used to assess the reliability of measured Nd isotopic ratios in studied grain-size fractions and bulk foraminiferal separates as provenance tracers. Rare earth element concentrations are reported in Fig. S2, normalized to the World River Average Silt (WRAS; *18*). Bulk foraminiferal separates exhibit shale-normalized patterns typical of seawater, exhibiting negative Ce anomalies and heavy-REE (HREE) enrichment relative to the light-REE (LREE). While foraminifers at Site U1482 show generally very good to excellent preservation down to 370 mbsf (*17*) and display similar shale-normalized patterns, the samples corresponding to the 4-8 Ma time interval (between ~165 and 370 mbsf) display less pronounced negative Ce anomalies (Ce/Ce* = 0.99 ± 0.09, 1 SD ; n=26) than those from the 0-4 Ma period (Ce/Ce* = 0.77 ± 0.17 ; n=20) (Fig. S2). This observation indicates that early diagenetic alteration progressively occurs with depth, accompanied by partial dissolution of biogenic calcite and Fe-Mn oxyhydroxide coatings, possibly followed by minor calcite overgrowth. Diagenetic alteration of foraminifers at Site U1482 is also associated with minor REE loss downcore, as inferred from slightly lower average Nd concentrations in the 4-to-8 Ma samples (Nd = 0.73 ± 0.23 µg/g, 1 SD; n=26) compared to the last 4 Ma period (Nd = 0.92 ± 0.21 µg/g; n=20) (Table S5). Importantly, the absence of any authigenic Nd addition in studied foraminiferal fractions provides reassuring evidence that they most likely still faithfully record the Nd isotopic composition of bottom waters at the time of sediment deposition (*22*).

Rationale for the exclusion of Nd isotope data in grain-size detrital fractions due to the presence of residual authigenic phases (secondary phosphate minerals)

At Site U1482, fine clays (<0.8 µm) from the 4-to-8 Ma time interval display relatively flat shale-normalized REE patterns, with Nd/Yb_N = 1.04 ± 0.13 (1 SD ; n=26) typical of river clays worldwide (*18*). In contrast, fine clays deposited over the last 4 Ma are characterized by slight LREE depletion relative to HREE (with Nd/Yb_N = 0.82 ± 0.09 ; n=20), i.e. a geochemical signature typical of volcanogenic sediments (*18*), hence consistent with the presence of mafic material transported by the Indonesian Throughflow (see Discussion in the main text). While most fine silts (4-12 µm) generally exhibit flat shale-normalized REE patterns, a few fine-silt samples and all medium-coarse silt fractions (10-63 µm) at Site U1482 display pronounced HREE enrichment relative to LREE (Fig. S2). These particular HREE-enriched patterns indicate the presence of heavy accessory minerals such as zircon (*18*), brought from nearby Australia via aaeolian transport. This is consistent with the evidence that the degree of HREE-enrichment in both fine and coarse silt fractions, as inferred from Nd/Yb_N, displays a negative correlation trend with Zr abundance (Fig. S3A). While the presence of zircon strongly influences the sedimentary REE budget of silt-size fractions at Site U1482, grain-size variability and the presence of accessory minerals has limited influence on Nd isotope ratios in fine-grained detrital sediments (*18*, *71*). Importantly, this means that the application of Nd isotopes to zircon-bearing silt-size fractions at Site U1482 can still provide reliable information on sediment provenance.

Another particular feature of our results is the evidence that a few silt-size fractions display markedly higher REE abundances than typical fine-grained sediments (Fig. S2). The fact that these particular REE-bearing samples are associated with mid-REE (MREE) enrichments indicates the presence of residual authigenic mineral phases, most likely phophates. Secondary REE-bearing phosphate minerals such as florencite typically form during early-diagenetic processes at continental margins. These minerals are ubiquitous components of shallow-marine sandstones of all ages in western Australian sedimentary basins, where they typically occur as small (<20 µm) discrete rhombohedral crystals or as overgrowths on quartz or zircon grains (*72, 73*). By inference, their presence in silt-size detrital fractions at Site U1482 probably relates to wind-blown inputs from nearby Australian sedimentary basins. Because these early-diagenetic REE-bearing minerals were initially formed in the marine environment, their Nd isotopic composition is likely to depart substantially from associated detrital mineral phases. This is illustrated in Fig. S3B by the fact that medium-coarse silt fractions characterized by high REE abundances (ΣREE_N) systematically display more radiogenic Nd isotope (ε_{Nd}) compositions. The presence of such residual REE-bearing authigenic phases in studied grain-size fractions could result in biased inferences on sediment provenance based on Nd isotopes. Therefore, all samples associated with anomalously high REE abundances and mid-REE enrichments (see the orange lines in Fig. S3) were excluded from the discussion based on Nd isotopes. This includes one fine clay fraction (1H1_145/150) ; eigth fine silt fractions (2H1, 3H1, 22H4, 26H4, 27H4, 28H4, 34H4, 37H4) ; and eight medium-coarse silt fractions (2H1, 3H1, 22H4, 23H4, 25H2, 26H4, 27H4, 34H4).

Quantifying relative source contributions using end-member mixing modeling

Quantitative constraints on past provenance changes at Site U1482 were obtained using mixing models combining ε_{Nd} and $(Nd/Yb)_N$ end-member compositions for northwest Australian felsic detrital inputs and Indonesian mafic sediments (Table S7). Because REE abundances in NW Australian detrital inputs are strongly influenced by the presence of zircons, separate end-members were defined for zircon-depleted (4-12 µm) and zircon-bearing (10-63 µm) silt-size fractions, both characterized by similar Nd isotopic compositions, but showing markedly differing $(Nd/Yb)_N$ ratios. Average end-member compositions for NW Australian sediment sources were determined using the mean ε_{Nd} and $(Nd/Yb)_N$ values for the 4.1 – 7.2 Ma time interval at Site U1482, hence integrating the long-term compositional variability observed between the 7.7-5.7 Ma arid period and the onset of more humid conditions after 5.1 Ma. End-member compositions for mafic sediment sources exported by the Indonesian Throughflow, presumably less likely to be influenced by grain-size effects, were estimated from values for marine and riverine fine-grained sediments (*21*, *69*, This study). Note that normalization values for REE abundances correspond to the World River Average Silt (WRAS; *18*).

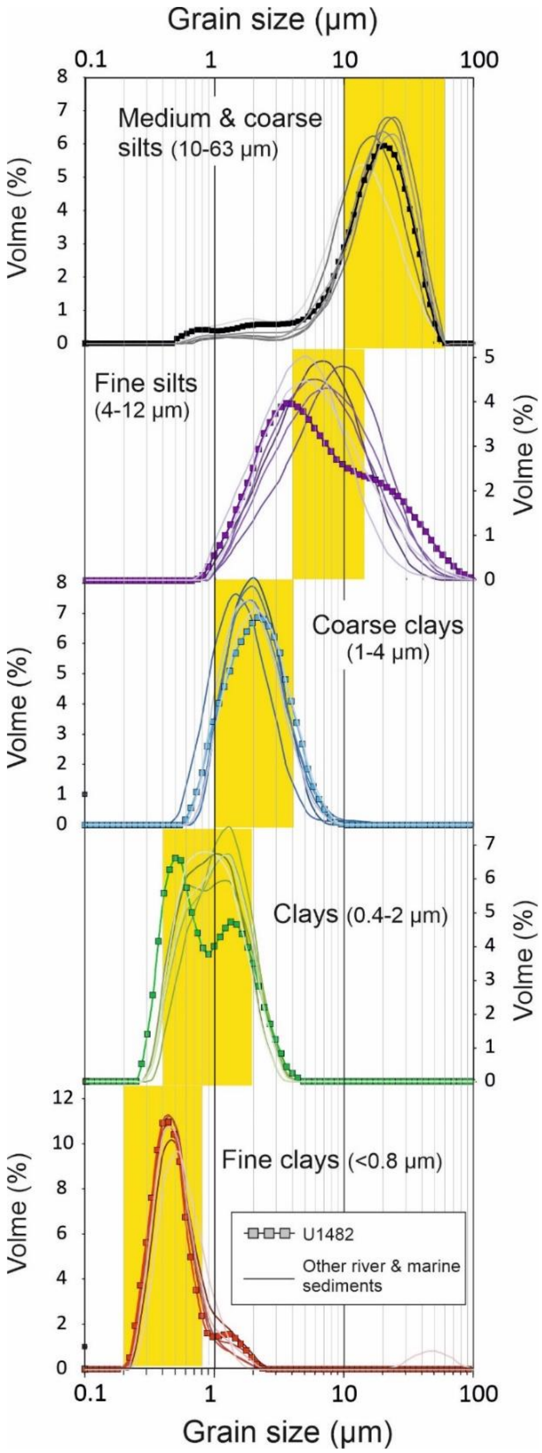

Fig. S1.

Particle-size analyses of detrital fractions separated by differential centrifugation from various river and marine fine-grained sediments.

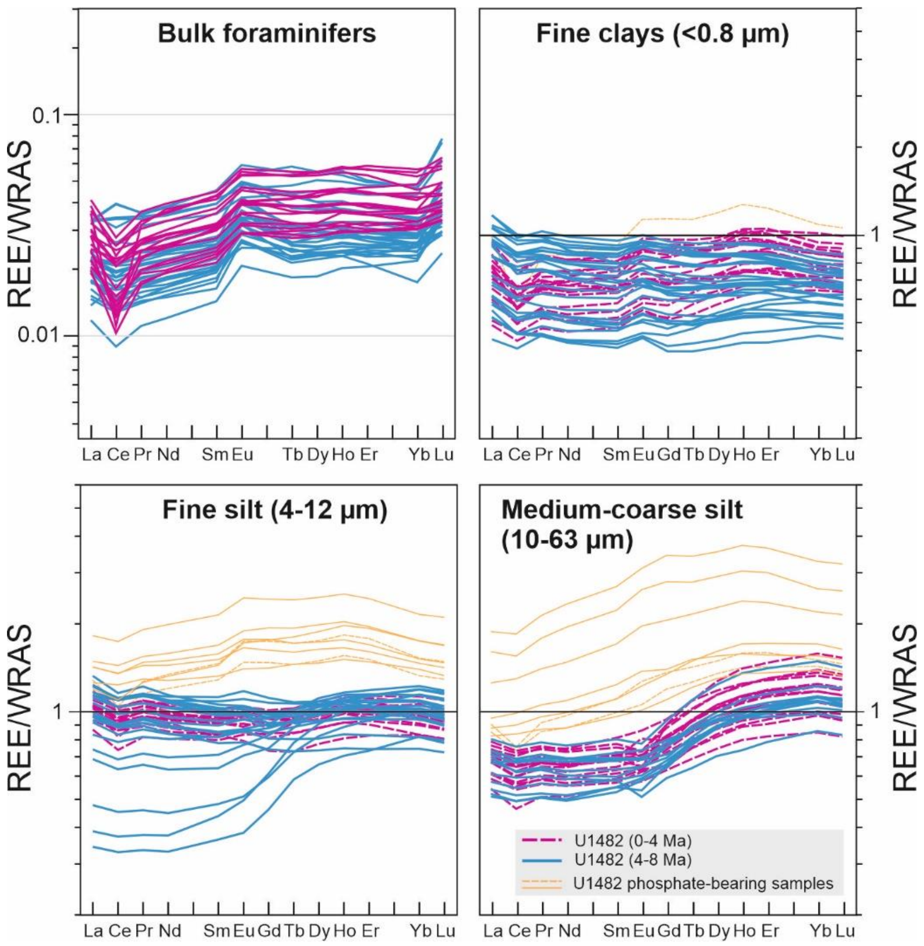

Fig. S2.

Shale-normalized REE patterns of studied grain-size detrital fractions and bulk foraminiferal separates at Site U1482. The shale reference values used for normalization correspond to the World River Average Silt (WRAS; *18*). The orange lines represent samples exhibiting relatively high REE abundances and mid-REE enrichments, which indicate the presence of early-diagenetic phosphate minerals presumably transported with aeolian dusts from nearby Australian sedimentary formations (*72*, *73*) and associated with distinctive Nd isotopic compositions (see Fig. S3B). In the manuscript, these samples were hence excluded from the discussion based on Nd isotopes.

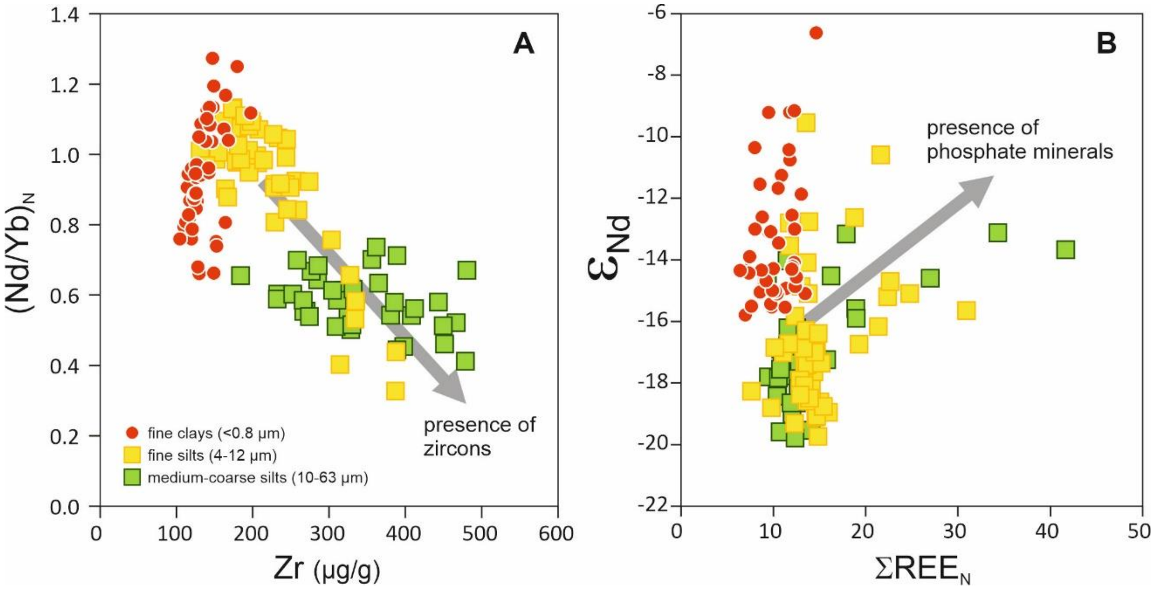

Fig. S3.

Relationship between $(Nd/Yb)_N$ vs. Zr (A), and ε_{Nd} vs. ΣREE_N (B) in studied grain-size detrital fractions at Site U1482. The subscript N refers to shale-normalized (WRAS; *18*). The observed correlation trends indicate the presence of silt-size zircon and early-diagenetic phosphate minerals brought by aeolian dusts from nearby Australia (*72*, *73*).

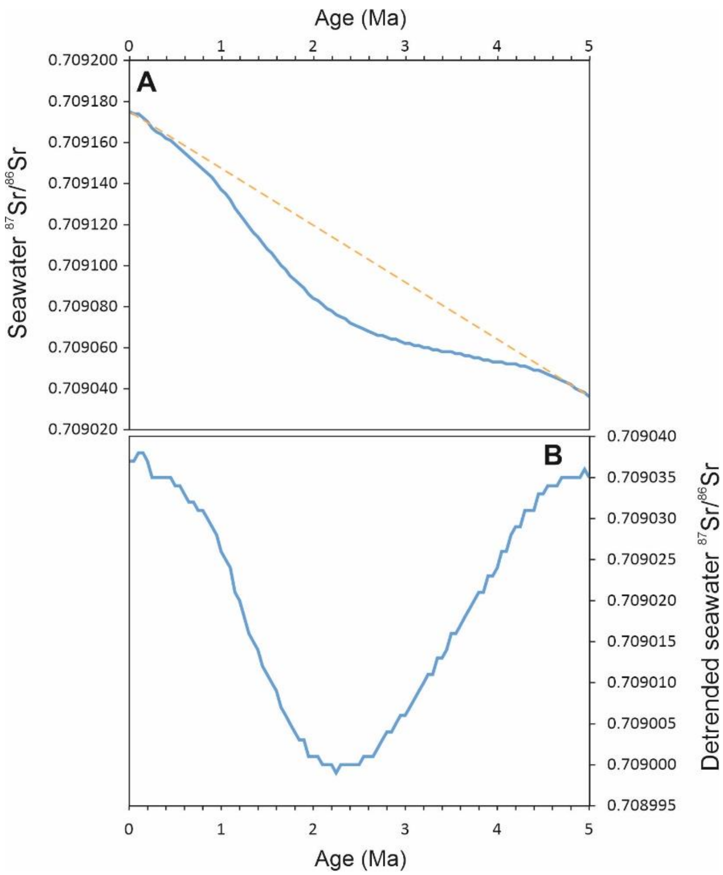

Fig. S4.

Late Neogene evolution of the marine Sr isotope curve. (A) Seawater $^{87}Sr/^{86}Sr$ curve for the last 5 Ma, showing a break in slope between ~4.5 and 2.5 Ma (*59*). Orange dashed line indicates the theoretical baseline Sr isotope curve used for numerical modeling. (B) Detrended seawater $^{87}Sr/^{86}Sr$ curve used to provide quantitative constraints on the riverine Sr flux from Indonesian islands required to explain the seawater $^{87}Sr/^{86}Sr$ 'plateau' between ~4.5 and 2.5 Ma.

Supplementary Tables captions

Table S1 : Mass accumulation rates of clay and silt-size fractions, and Nd isotopic compositions of foraminiferal separates and grain-size detrital fractions at Site U1482

Table S2 : Rare earth and trace element abundances in fine clay (<0.8µm) detrital fractions at Site U1482

Table S3 : Rare earth and trace element abundances in fine silt (4-12 µm) detrital fractions at Site U1482

Table S4 : Rare earth and trace element abundances in coarse silt (10-63 µm) detrital fractions at Site U1482

Table S5 : Rare earth and trace element abundances in bulk foraminiferal separates (>125 µm) at Site U1482

Table S6 : Nd isotopic compositions of fine-grained detrital river sediments in Northwestern Australia and the Indonesian region

Table S7 : Three-component mixing models: Nd isotope and $(Nd/Yb)_N$ values of detrital end-members.

Table S8 : Parameters used for mass balance modeling of seawater Sr isotopes.

REFERENCES AND NOTES

1. Y. Dilek, H. Furnes, Ophiolite genesis and global tectonics: Geochemical and tectonic fingerprinting of ancient oceanic lithosphere. *GSA Bulletin* **123**, 387–411 (2011).

2. O. Jagoutz, F. A. Macdonald, L. Royden, Low-latitude arc–Continent collision as a driver for global cooling. *Proc. Natl. Acad. Sci. U.S.A.* **113**, 4935–4940 (2016).

3. F. A. Macdonald, N. L. Swanson-Hysell, Y. Park, L. Lisiecki, O. Jagoutz, Arc-continent collisions in the tropics set Earth's climate state. *Science* **364**, 181–184 (2019).

4. J. Hartmann, N. Moosdorf, R. Lauerwald, M. Hinderer, A. J. West, Global chemical weathering and associated P-release—The role of lithology, temperature and soil properties. *Chem. Geol.* **363**, 145–163 (2014).

5. N. L. Swanson-Hysell, F. A. Macdonald, Tropical weathering of the Taconic orogeny as a driver for Ordovician cooling. *Geology* **45**, 719–722 (2017).

6. M. Pubellier, C. Monnier, R. C. Maury, R. Tamayo, Plate kinematics, origin and tectonic emplacement of supra-subduction ophiolites in SE Asia. *Tectonophysics* **392**, 9–36 (2004).

7. R. Hall, Cenozoic geological and plate tectonic evolution of SE Asia and the SW Pacific: Computer-based reconstructions, model and animations. *J. Asian Earth Sci.* **20**, 353–431 (2002).

8. J. D. Milliman, K. L. Farnsworth, *River Discharge to the Coastal Ocean: A Global Synthesis* (Cambridge Univ. Press, 2013).

9. P. Molnar, T. W. Cronin, Growth of the Maritime Continent and its possible contribution to recurring Ice Ages. *Paleoceanography* **30**, 196–225 (2015).

10. Y. Park, P. Maffre, Y. Goddéris, F. A. Macdonald, E. S. C. Anttila, N. L. Swanson-Hysell, Emergence of the Southeast Asian islands as a driver for Neogene cooling. *Proc. Natl. Acad. Sci. U.S.A.* **117**, 25319–25326 (2020).

11. S. Zhang, N. J. Planavsky, The silicate weathering feedback in the context of ophiolite emplacement: Insights from an inverse model of global weathering proxies. *Am. J. Sci.* **319**, 75–104 (2019).

12. J. K. C. Rugenstein, D. E. Ibarra, S. Zhang, N. J. Planavsky, F. von Blanckenburg, Isotope mass-balance constraints preclude that mafic weathering drove Neogene cooling. *Proc. Natl. Acad. Sci. U.S.A.* **118**, e2026345118 (2021).

13. M. A. Cane, P. Molnar, Closing of the Indonesian seaway as a precursor to east African aridification around 3–4 million years ago. *Nature* **411**, 157–162 (2001).

14. C. Karas, D. Nürnberg, A. K. Gupta, R. Tiedemann, K. Mohan, T. Bickert, Mid-Pliocene climate change amplified by a switch in Indonesian subsurface Throughflow. *Nat. Geosci.* **2**, 434–438. (2009).

15. W. Kuhnt, A. Holbourn, R. Hall, M. Zuvela, R. Käse, Neogene history of the Indonesian throughflow. *Continent-Ocean Interactions within East Asian Marginal Seas, Geophys. Monogr.*, Vol. 149, 299–320 (2004).

16. D. De Vleeschouwer, G. Auer, R. Smith, K. Bogus, B. Christensen, J. Groeneveld, B. Petrick, J. Henderiks, I. S. Castañeda, E. O'Brien, M. Ellinghausen, S. J. Gallagher, C. S. Fulthorpe, H. Pälike, The amplifying effect of Indonesian Throughflow heat transport on Late Pliocene Southern Hemisphere climate cooling. *Earth Planet. Sci. Lett.* **500**, 15–27 (2018).

17. Y. Rosenthal, A. Holbourn, D. Kulhanek, Expedition 363 Scientists, Expedition 363 preliminary report: Western Pacific warm pool. *Proc. Integr. Ocean Drill. Program, Exped. 363* (2017); http://dx.doi.org/10.14379/iodp.pr.363.2017.

18. G. Bayon, S. Toucanne, C. Skonieczny, L. André, S. Bermell, S. Cheron, B. Dennielou, J. Etoubleau, N. Freslon, T. Gauchery, Y. Germain, S. J. Jorry, G. Ménot, L. Monin, E. Ponzevera, M. L. Rouget, K. Tachikawa, J. A. Barrat, Rare earth elements and neodymium isotopes in world river sediments revisited. *Geochim. Cosmochim. Acta* **170**, 17–38 (2015).

19. K. C. Condie, Chemical composition and evolution of the upper continental crust: Contrasting results from surface samples and shales. *Chem. Geol.* **104**, 1–37 (1993).

20. F. X. Gingele, P. De Deckker, C. D. Hillenbrand, Clay mineral distribution in surface sediments between Indonesia and NW Australia—Source and transport by ocean currents. *Mar. Geol.* **179**, 135–146 (2001).

21. C. Ehlert, M. Frank, B. A. Haley, U. Böniger, P. De Deckker, F. X. Gingele, Current transport versus continental inputs in the eastern Indian Ocean: Radiogenic isotope signatures of clay size sediments. *Geochem. Geophys. Geosyst.* **12**, Q06017 (2011).

22. K. Tachikawa, A. M. Piotrowski, G. Bayon, Neodymium associated with foraminiferal carbonate as a recorder of seawater isotopic signatures. *Quat. Sci. Rev.* **88**, 1–13 (2014).

23. B. A. Christensen, W. Renema, J. Henderiks, D. de Vleeschouwer, J. Groeneveld, I. S. Castañeda, L. Reuning, K. Bogus, G. Auer, T. Ishiwa, C. M. McHugh, S. J. Gallagher, C. S. Fulthorpe, IODP Expedition 356 Scientists, Indonesian Throughflow drove Australian climate from humid Pliocene to arid Pleistocene. *Geophys. Res. Lett.* **44**, 6914–6925. (2017).

24. J. B. W. Stuut, P. De Deckker, M. Saavedra-Pellitero, F. Bassinot, A. J. Drury, M. H. Walczak, K. Nagashima, M. Murayama, A 5.3-million-year history of monsoonal precipitation in northwestern Australia. *Geophys. Res. Lett.* **46**, 6946–6954 (2019).

25. R. Pei, W. Kuhnt, A. Holbourn, J. Hingst, M. Koppe, J. Schultz, P. Kopetz, P. Zhang, N. Andersen, Monitoring Australian Monsoon variability over the past four glacial cycles. *Palaeogeogr. Palaeoclimatol. Palaeoecol.* **568**, 110280 (2021).

26. C. Jeandel, D. Thouron, M. Fieux, Concentrations and isotopic compositions of neodymium in the eastern Indian Ocean and Indonesian straits. *Geochim. Cosmochim. Acta* **62**, 2597–2607 (1998).

27. S. Le Houedec, L. Meynadier, C. J. Allègre, Nd isotope systematics on ODP Sites 756 and 762 sediments reveal major volcanic, oceanic and climatic changes in South Indian Ocean over the last 35 Ma. *Earth Planet. Sci. Lett.* **327–328**, 29–38 (2012).

28. K. Wallmann, G. Aloisi, M. Haeckel, P. Tishchenko, G. Pavlova, J. Greinert, S. Kutterolf, A. Eisenhauer, Silicate weathering in anoxic marine sediments. *Geochim. Cosmochim. Acta* **72**, 2895–2918 (2008).

29. M. E. Torres, W. L. Hong, E. A. Solomon, K. Milliken, J. H. Kim, J. C. Sample, B. M. A. Teichert, K. Wallmann, Silicate weathering in anoxic marine sediment as a requirement for authigenic carbonate burial. *Earth Sci. Rev.* **200**, 102960 (2020).

30. J. Du, B. A. Haley, A. C. Mix, A. N. Abbott, J. McManus, D. Vance, Reactive-transport modeling of neodymium and its radiogenic isotope in deep-sea sediments: The roles of authigenesis, marine silicate weathering and reverse weathering. *Earth Planet. Sci. Lett.* **596**, 117792 (2022).

31. C. R. Pearce, M. T. Jones, E. H. Oelkers, C. Pradoux, C. Jeandel, The effect of particulate dissolution on the neodymium (Nd) isotope and Rare Earth Element (REE) composition of seawater. *Earth Planet. Sci. Lett.* **369–370**, 138–147 (2013).

32. G. Auer, D. de Vleeschouwer, R. A. Smith, K. Bogus, J. Groeneveld, P. Grunert, I. S. Castañeda, B. Petrick, B. Christensen, C. Fulthorpe, S. J. Gallagher, J. Henderiks, Timing and pacing of Indonesian Throughflow restriction and its connection to Late Pliocene climate shifts. *Paleoceanogr. Paleoclimatol.* **34**, 635–657. (2019).

33. R. Stumpf, S. Kraft, M. Frank, B. Haley, A. Holbourn, W. Kuhnt, Persistently strong Indonesian Throughflow during marine isotope stage 3: Evidence from radiogenic isotopes. *Quat. Sci. Rev.* **112**, 197–206 (2015).

34. R. Hall, Southeast Asia's changing palaeogeography. *Blumea* **54**, 148–161 (2009).

35. R. Hall, Southeast Asia: New views of the geology of the Malay Archipelago. *Annu. Rev. Earth Planet. Sci.* **45**, 331–358 (2017).

36. S. L. Baldwin, P. G. Fitzgerald, L. E. Webb, Tectonics of the New Guinea region. *Annu. Rev. Earth Planet. Sci.* **40**, 495–520 (2012).

37. N. Roosmawati, R. Harris, Surface uplift history of the incipient Banda arc-continent collision: Geology and synorogenic foraminifera of Rote and Savu Islands, Indonesia. *Tectonophysics* **479**, 95–110 (2009).

38. N. Nguyen, B. Duffy, J. Shulmeister, M. Quigley, Rapid Pliocene uplift of Timor. *Geology* **41**, 179–182 (2013).

39. R. Harris, Rise and fall of the Eastern Great Indonesian arc recorded by the assembly, dispersion and accretion of the Banda Terrane, Timor. *Gondwana Res.* **10**, 207–231 (2006).

40. A. M. S. Nugraha, R. Hall, Late Cenozoic palaeogeography of Sulawesi, Indonesia. *Palaeogeogr. Palaeoclimatol. Palaeoecol.* **490**, 191–209 (2018).

41. R. Hall, M. G. Audley-Charles, F. T. Banner, S. Hidayat, S. L. Tobing, Late Palaeogene–Quaternary geology of Halmahera, Eastern Indonesia: Initiation of a volcanic island arc. *J. Geol. Soc.* **145**, 577–590 (1988).

42. A. A. Pairault, R. Hall, C. F. Elders, Structural styles and tectonic evolution of the Seram Trough, Indonesia. *Mar. Petrol. Geol.* **20**, 1141–1160 (2003).

43. R. Hall, G. Nichols, Cenozoic sedimentation and tectonics in Borneo: Climatic influences on orogenesis. *Geol. Soc. London Spec. Pub.* **191**, 5–22 (2002).

44. C. K. Morley, S. Back, Estimating hinterland exhumation from late orogenic basin volume, NW Borneo. *J. Geol. Soc.* **165**, 353–366 (2008).

45. R. Hall, Australia–SE Asia collision: Plate tectonics and crustal flow. *Geol. Soc. London Spec. Pub.* **355**, 75–109 (2011).

46. M. W. Wara, A. C. Ravelo, M. L. Delaney, Permanent El Niño-like conditions during the Pliocene warm period. *Science* **309**, 758–761 (2005).

47. A. V. Fedorov, N. J. Burls, K. T. Lawrence, L. C. Peterson, Tightly linked zonal and meridional sea surface temperature gradients over the past five million years. *Nat. Geosci.* **8**, 975–980 (2015).

48. J. Liu, J. Tian, Z. Liu, T. D. Herbert, A. V. Fedorov, M. Lyle, Eastern equatorial Pacific cold tongue evolution since the late Miocene linked to extratropical climate. *Sci. Adv.* **5**, eaau6060 (2019).

49. X. Liu, M. Huber, G. L. Foster, A. Dessler, Y. G. Zhang, Persistent high latitude amplification of the Pacific Ocean over the past 10 million years. *Nat. Commun.* **13**, 7310 (2022).

50. A. C. Ravelo, P. S. Dekens, M. McCarthy, Evidence for El Niño-like conditions during the Pliocene. *GSA Today* **16**, 3 (2006).

51. K. E. Dayem, D. C. Noone, P. Molnar, Tropical western Pacific warm pool and maritime continent precipitation rates and their contrasting relationships with the Walker Circulation. *J. Geophys. Res. Atmos.* **112**, (2007).

52. N. Peng, H. Dang, J. Wu, I. W. Aiello, Z. Jian, Tectonic and climatic controls on the Plio-Pleistocene evolution of sediment discharge from Papua New Guinea. *Mar. Geol.* **441**, 106627 (2021).

53. P. Molnar, P. England, Late Cenozoic uplift of mountain ranges and global climate change: Chicken or egg? *Nature* **346**, 29–34 (1990).

54. J. K. Caves Rugenstein, D. E. Ibarra, F. von Blanckenburg, Neogene cooling driven by land surface reactivity rather than increased weathering fluxes. *Nature* **571**, 99–102 (2019).

55. M. E. Raymo, W. F. Ruddiman, Tectonic forcing of late Cenozoic climate. *Nature* **359**, 117–122 (1992).

56. B. Peucker-Ehrenbrink, G. Ravizza, A. W. Hofmann, The marine $^{187}Os/^{186}Os$ record of the past 80 million years. *Earth Planet. Sci. Lett.* **130**, 155–167 (1995).

57. L. Oliver, N. Harris, M. Bickle, H. Chapman, N. Dise, M. Horstwood, Silicate weathering rates decoupled from the $^{87}Sr/^{86}Sr$ ratio of the dissolved load during Himalayan erosion. *Chem. Geol.* **201**, 119–139 (2003).

58. M. A. Torres, A. J. West, K. E. Clark, G. Paris, J. Bouchez, C. Ponton, S. J. Feakins, V. Galy, J. F. Adkins, The acid and alkalinity budgets of weathering in the Andes–Amazon system: Insights into the erosional control of global biogeochemical cycles. *Earth Planet. Sci. Lett.* **450**, 381–91 (2016).

59. J. M. McArthur, R. J. Howarth, G. A. Shields, Strontium Isotope Stratigraphy, in *The Geologic Time Scale 2012*, F. M. Gradstein, J. G. Ogg, M. Schmitz, G. Ogg, Eds. (Elsevier, 2012), pp. 127–144.

60. D. A. Hodell, G. A. Mead, P. A. Mueller, Variation in the strontium isotopic composition of seawater (8 Ma to present): Implications for chemical weathering rates and dissolved fluxes to the oceans. *Chem. Geol.* **80**, 291–307 (1990).

61. B. Peucker-Ehrenbrink, G. J. Fiske, A continental perspective of the seawater $^{87}Sr/^{86}Sr$ record: A review. *Chem. Geol.* **510**, 140–165 (2019).

62. W. L. Hong, M. E. Torres, S. Kutterolf, Towards a global quantification of volcanogenic aluminosilicate alteration rates through the mass balance of strontium in marine sediments. *Chem. Geol.* **550**, 119743 (2020).

63. N. R. McKenzie, B. K. Horton, S. E. Loomis, D. F. Stockli, N. J. Planavsky, C. T. A. Lee, Continental arc volcanism as the principal driver of icehouse-greenhouse variability. *Science* **352**, 444–447 (2016).

64. T. D. Herbert, C. A. Dalton, Z. Liu, A. Salazar, W. Si, D. S. Wilson, Tectonic degassing drove global temperature trends since 20 Ma. *Science* **377**, 116–119 (2022).

65. G. Bayon, C. R. German, R. M. Boella, J. A. Milton, R. N. Taylor, R. W. Nesbitt, An improved method for extracting marine sediment fractions and its application to Sr and Nd isotopic analysis. *Chem. Geol.* **187**, 179–199 (2002).

66. A. Bouvier, J. D. Vervoort, P. J. Patchett, The Lu–Hf and Sm–Nd isotopic composition of CHUR: Constraints from unequilibrated chondrites and implications for the bulk composition of terrestrial planets. *Earth Planet. Sci. Lett.* **273**, 48–57 (2008).

67. J. C. Walker, P. B. Hays, J. F. Kasting, A negative feedback mechanism for the long-term stabilization of Earth's surface temperature. *J. Geophys. Res.* **86**, 9776–9782 (1981).

68. P. De Deckker, An evaluation of Australia as a major source of dust. *Earth Sci. Rev.* ***194***, 536–567 (2019).

69. G. Bayon, T. Lambert, N. Vigier, P. De Deckker, N. Freslon, K. Jang, C. S. Larkin, A. M. Piotrowski, K. Tachikawa, M. Thollon, E. T. Tipper, Rare earth element and neodymium isotope tracing of sedimentary rock weathering. *Chem. Geol.* **553**, 119794 (2020).

70. S. Krumm, SediCalc (free geological software). GeoZentrum Nordbayern, Universität Erlangen-Nürnberg (2006); http://sedicalc.sourceforge.net.

71. T. N. Jonell, Y. Li, J. Blusztajn, L. Giosan, P. D. Clift, Signal or noise? Isolating grain size effects on Nd and Sr isotope variability in Indus delta sediment provenance. *Chem. Geol.* **485**, 56–73 (2018).

72. B. Rasmussen, Early-diagenetic REE-phosphate minerals (florencite, gorceixite, crandallite, and xenotime) in marine sandstones; a major sink for oceanic phosphorus. *Am. J. Sci.* **296**, 601–632 (1996).

73. B. Rasmussen, R. Buick, W. R. Taylor, Removal of oceanic REE by authigenic precipitation of phosphatic minerals. *Earth Planet. Sci. Lett.* **164**, 135–149 (1998).

74. K. Wallmann, Controls on the Cretaceous and Cenozoic evolution of seawater composition, atmospheric CO_2 and climate. *Geochim. Cosmochim. Acta* **65**, 3005–3025. (2001).

75. M. R. Palmer, J. M. Edmond, The strontium isotope budget of the modern ocean. *Earth Planet. Sci. Lett.* **92**, 11–26 (1989).

76. F. M. Richter, D. B. Rowley, D. J. DePaolo, Sr isotope evolution of seawater: The role of tectonics. *Earth Planet. Sci. Lett.* **109**, 11–23 (1992).
